# JPH3 Facilitates Cisplatin Resistance in Anaplastic Thyroid Cancer via Activation of the JAK–STAT Signaling Pathway

**DOI:** 10.1002/cam4.71750

**Published:** 2026-03-20

**Authors:** Xiuyan Wang, Sha Luan, Lei Sun, Lixin Lian, Ming Qi

**Affiliations:** ^1^ The Fourth Department of General Surgery The First Hospital of Harbin Harbin Heilongjiang China; ^2^ Department of Nuclear Medicine The Fourth Affiliated Hospital of Harbin Medical University Harbin Heilongjiang China; ^3^ Department of Critical Care Medicine The First Hospital of Harbin Harbin Heilongjiang China

**Keywords:** anaplastic thyroid cancer, COL26A1, DDP resistance, JAK–STAT signaling pathway, JPH3, thyroid cancer

## Abstract

**Background:**

Junctophilin 3 (JPH3) acts as a tumor suppressor in several cancers; however, the role of JPH3 in anaplastic thyroid cancer (ATC) is still unknown.

**Methods:**

Via bioinformatics prediction and verified through Western blot experiments, the expression level of JPH3 in ATC was ascertained. Subsequently, the role of JPH3 in ATC cells was validated via in vitro and in vivo experiments, and the molecular mechanism of JPH3 in ATC was further illuminated. Eventually, the mechanism of JPH3 on cisplatin (DDP)‐resistant ATC cells was probed. The results indicated that JPH3 was highly expressed in thyroid cancer (TC), and the survival period of patients with high expression was significantly curtailed.

**Results:**

JPH3 was upregulated in TC tissues compared with the normal thyroid tissues. Our experiments disclosed that JPH3 acted as an oncogene in ATC cells, facilitating tumor development, and JPH3 can activate the JAK–STAT signaling pathway by upregulation of collagen type XXVI alpha 1 chain (COL26A1), and experiments attested that JPH3 promoted the proliferation, invasion, and migration of ATC cells by activating the JAK–STAT signaling pathway. Further research uncovered that JPH3 was also involved in the process of DDP resistance in ATC cells. By activating the JAK–STAT signaling pathway, JPH3 induced the malignant phenotype of DDP‐resistant ATC cells.

**Conclusions:**

JPH3 functions as a potent oncogenic driver in ATC, promoting tumor proliferation, invasion, and migration through the activation of the JAK–STAT signaling pathway. This oncoprotein contributes to enhanced DDP resistance in ATC cells via JAK–STAT‐mediated mechanisms, offering novel insights into the molecular basis of chemoresistance in this highly aggressive malignancy.

## Graphical abstract

JPH3 upregulation promotes tumorigenesis in anaplastic thyroid carcinoma (ATC) via activation of the COL26A1‐dependent JAK–STAT signaling pathway; sustained JPH3 overexpression further amplifies signaling through this axis, thereby conferring cisplatin (DDP) resistance.

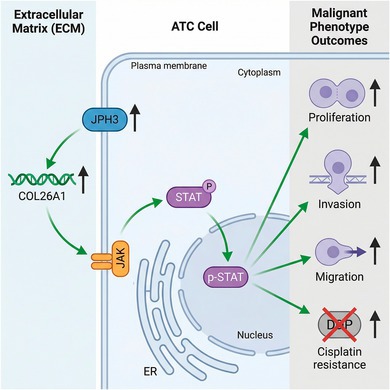

## 1 Introduction

Thyroid cancer (TC) represents a frequently encountered endocrine malignancy in clinical scenarios, with its incidence steadily escalating due to enhancements in diagnostic methodologies [1, 2, 3]. TC is primarily categorized into papillary thyroid carcinoma (PTC) and anaplastic thyroid cancer (ATC). Although ATC is less common, it is characterized by a markedly low degree of differentiation and an exceptionally high level of malignancy, positioning it among the deadliest cancers [4]. The average overall survival for affected individuals is approximately 3 months. Cisplatin (DDP) is a commonly employed chemotherapeutic agent; however, the development of DDP resistance is a critical factor that exacerbates the poor prognosis and diminishes the survival rates of patients with ATC [5]. Thus, a comprehensive understanding of the molecular pathways involved in the progression of ATC, particularly those associated with drug resistance, is essential for formulating innovative treatment strategies for this highly aggressive malignancy.

Junctophilin 3 (JPH3) is a vital constituent of the junctional membrane complex (JMC) protein family. It plays a significant role in maintaining the stability of the junctional membrane complex that exists between the endoplasmic reticulum (ER) and the plasma membrane (PM), as well as preserving the cellular architecture that connects the cell surface with intracellular ion channels [6]. Alterations in JPH proteins have been linked to a variety of human disorders, including cardiomyopathy, arrhythmia, heart failure, myopathy, and diseases resembling Huntington's disease [7]. Currently, JPH3 is recognized for its function as an anti‐oncogene across multiple tumor types. For instance, research has indicated that JPH3 exhibits low expression levels in gastrointestinal tumors [8] and hepatocellular carcinoma (HCC) [9], with higher expression of JPH3 correlating with diminished tumor proliferation. Furthermore, a bioinformatics assessment of the Gene Expression Omnibus (GEO) database revealed elevated JPH3 expression in glioma tissues, where patients displaying high JPH3 levels experienced extended survival periods [10]. Conversely, an analysis of the TCGA database employing the DO‐UniBIC approach revealed that JPH3 was overexpressed in breast cancer, with patients showing high JPH3 levels suffering from shorter survival durations, suggesting that JPH3 may function as an oncogene in this context [11]. In ATC, our bioinformatics analysis corroborated findings observed in breast cancer; however, the mechanisms governing JPH3 expression in ATC warrant further investigation.

In this study, the expression levels of JPH3 in TC were evaluated through bioinformatics approaches. Its functional role in ATC was subsequently validated via both in vivo and in vitro experiments. The findings reveal that JPH3 facilitates the progression of ATC through the activation of the JAK–STAT3 signaling pathway. Notably, it was demonstrated that JPH3 can augment the resistance of ATC to DDP via the JAK/STAT3 pathway, offering new insights into the molecular mechanisms underlying DDP resistance in ATC.

## 2 Materials and Methods

### 2.1 Tissue Samples

Thirty pairs of ATC and adjacent normal thyroid tissues were procured from Harbin First Hospital. Immediately following excision, the surgical samples were rapidly frozen in liquid nitrogen. The pathological diagnosis confirmed ATC. The patients had not received any form of chemotherapy or radiation therapy before surgery. This research was approved by the ethics committee of Harbin First Hospital, and all participants provided informed consent before their involvement.

### 2.2 Cell Culture

Human ATC cell lines CAL‐62, BHT101, 8505C, and KMH‐5 M were acquired from Wuhan Procell Life Science Technology Co. Ltd. (Wuhan, China). The immortalized human thyroid follicular epithelial cell line, designated as hy‐ori3‐1, was acquired from the Bena Culture Collection located in Suzhou, China. These cells were cultured in a high‐glucose DMEM medium with the addition of 10% fetal bovine serum (FBS, sourced from Biosharp, China), along with 100 U/mL of penicillin (Solarbio Life Sciences, Beijing, China) and 100 μg/mL of streptomycin (Solarbio Life Sciences, Beijing, China) in an incubator set at 37°C with 5% CO_2_. All cell lines used in this study were independently authenticated by short tandem repeat (STR) profiling by experts before the experiment to ensure their identity and purity. All cell lines used in this study were confirmed to be free of mycoplasma contamination.

### 2.3 Cell Transfection

For cell transfection, Lipofectamine 3000 (Catalog No.: #L3000150, Thermo Fisher Scientific, Waltham, MA, USA) was utilized following the manufacturer's guidelines. Unless otherwise stated, all subsequent functional experiments were performed using transient transfection.

### 2.4 Generation of DDP‐Resistant Cells

To generate DDP‐resistant cell lines, logarithmically growing CAL‐62 and BHT101 cells were selected and cultured in DMEM supplemented with DDP. The concentration of DDP was incrementally increased in a stepwise manner (0.1, 0.2, 0.4, 0.8, 1.0 μg/mL DDP) until cells capable of withstanding 1.0 μg/mL DDP were established, specifically the CAL‐62/DDP and BHT101/DDP cells. The sensitivity of these cells to varying DDP concentrations was evaluated using the CC‐8 assay, with the inhibition rate calculated as (OD450 of the drug‐treated group/OD450 of the untreated group) × 100%.

### 2.5 Real‐Time Quantitative Polymerase Chain Reaction

For real‐time quantitative polymerase chain reaction (RT‐qPCR), total RNA was extracted in accordance with the TRIzol reagent kit protocols, followed by reverse transcription into cDNA using the TransScript First‐Strand cDNA Synthesis Super Mix for RT‐PCR. The PCR reactions were conducted as per the instructions for the TransStart Green qPCR Super Mix reagent kit. The primer sequences used are as follows: JPH3 Forward Primer: 5′‐CGACGGAGGGTCCTACTGT‐3′, JPH3 Reverse Primer: 5′‐AGCCGGTGTATTCGCCTTG‐3′; GAPDH Forward Primer: 5′‐ACAACTTTGGTATCGTGGAAGG‐3′, GAPDH Reverse Primer: 5′‐GCCATCACGCCACAGTTTC‐3′. The quantitative PCR was performed using the ABI QuantStudio 6 instrument, employing GAPDH as an internal control. Data analysis was conducted utilizing the 2^‐△△ct^ method.

### 2.6 CCK‐8 Assay

In the CCK‐8 assay, ATC cells in logarithmic growth phase were harvested at a density of 3 × 10^3^ cells per well and plated into 96‐well plates. Following the seeding, CCK‐8 reagent was added at 24 h, 48 h, and 72 h, with a further 2 h incubation before measurement of optical density (OD) at 450 nm using a microplate reader.

### 2.7 Colony Formation Assay

In the colony formation assay, 1 × 10^3^ ATC cells were plated into 6‐well plates and cultured for 2 weeks. Then, the cells were fixed with paraformaldehyde and stained with crystal violet. The number of clones formed by the cells was counted using Image J software.

### 2.8 Transwell Assay

Transwell assays were utilized to evaluate the migratory and invasive potential of the cells. For migration assessment, the Transwell chamber was used without Matrigel; conversely, in evaluating invasion capability, 100 μL of Matrigel at a concentration of 1 mg/mL was applied to the bottom layer of the Transwell chamber and allowed to gel at 37°C. In the upper compartment of the Transwell, 200 μL of cell suspension (1 × 10^4^ cells for migration, 4 × 10^4^ cells for invasion) was introduced, and the cells were incubated in a 5% CO_2_ environment at 37°C for 48 h. Upon removal of the Transwell, the chamber was washed with PBS, fixed with 70% ice‐cold ethanol for 1 h, stained, and allowed to sit at room temperature for 20 min. The chamber was then washed with PBS, and the non‐migratory cells on the upper compartment's surface were carefully wiped off using a contamination‐free cotton swab under a microscope.

### 2.9 In Vivo Animal Experiments

For in vivo experiments, CAL‐62 cells stably expressing JPH3 (3 × 10^6^ cells in 0.1 mL of phosphate‐buffered saline) were injected into the dorsal side of the right flank of 6‐week‐old female nude mice (with six mice per group). Tumor volume was measured every other day for a duration of 3 weeks. The tumor volume was calculated using the formula: (short diameter)^2^ × (long diameter)/2. After 3 weeks, the mice were euthanized, and the subcutaneous tumors were excised and weighed, and subjected to subsequent experiments.

### 2.10 Venn Diagram Analysis

RNA‐seq data related to TC were retrieved from The Cancer Genome Atlas (TCGA) database. Differential expression analysis was performed using the DESeq2 R package. Genes with an absolute log2 fold change greater than 1 and an adjusted *p* value (padj) less than 0.05 were considered statistically significant and were identified as differentially expressed genes (DEGs). Samples were stratified into high‐ and low‐expression groups based on JPH3 expression levels, using the median value as the cutoff. Spearman's rank correlation analysis was performed to identify genes significantly associated with JPH3 expression, with a threshold of absolute correlation coefficient > 0.3 and false discovery rate (FDR)–adjusted *p* value < 0.05. Subsequently, differential expression analysis was conducted using the DESeq2 package in R, comparing the two groups to identify DEGs with an absolute log2 fold change > 1 and FDR‐adjusted *p* value < 0.05. Survival analysis was performed using the survival R package in R software. Genes with a hazard ratio (HR) < 1 and a statistically significant *p* value (< 0.05) were selected for subsequent analysis.

Venn diagram analysis was performed using an online tool (http://bioinformatics.psb.ugent.be/webtools/Venn/).

### 2.11 Western Blot

The extraction of total proteins from cells was performed using RIPA lysis buffer supplemented with protease inhibitors, followed by quantification of protein concentration employing the BCA assay. After quantification, the proteins were subjected to denaturation, separated through gel electrophoresis, and subsequently transferred onto a PVDF membrane (Catalog No.: # 0000187216, 0.45 μm, Millipore Corp., Burlington, MA, USA). The membrane was then incubated overnight at 4°C with the designated primary antibodies. The following day, the membrane was treated with HRP‐conjugated goat anti‐rabbit or mouse secondary antibodies, after which an ECL luminescence solution was applied, and imaging was conducted utilizing a gel imaging analysis system. Grayscale analysis of the resulting images was performed with ImageJ software. The primary antibodies utilized in this study included: Anti‐JPH3 antibody (1:500, Catalog No.: ab79063, Abcam, Boston, MA, USA), β‐tubulin (Catalog No: 10068–1‐AP, 1:5000, Proteintech, China), Anti‐E‐cadherin antibody (1:1000, Catalog No.: ab231303, Abcam, Boston, MA, USA), Anti‐Vimentin antibody (1:5000, Catalog No.: ab92547, Abcam, Boston, MA, USA), Anti‐JAK2 antibody (1:5000, Catalog No.: ab108596, Abcam, Boston, MA, USA), Anti‐p‐JAK2 antibody (1:1000, Catalog No.: ab32101, Abcam, Boston, MA, USA), Anti‐STAT3 antibody (1:1000, Catalog No.: #9139, CST, Boston, MA, USA), and Anti‐p‐STAT3 antibody (1:1000, Catalog No.: #9139, CST, Boston, MA, USA), Anti‐COL6A1 antibody (1:1000, Catalog No.: ab151422, Abcam, Boston, MA, USA).

### 2.12 Immunohistochemistry

Immunohistochemistry (IHC) staining was employed to evaluate the expression levels of JPH3 (1:100, Catalog No.: bs‐11083R, Bioss, Beijing, China) and Ki67 (1:500, Catalog No.: bsm‐52455R, Bioss, Beijing, China) in paraffin‐embedded tissue samples, adhering to previously established IHC protocols.

### 2.13 Statistical Analyses

Statistical evaluations were conducted utilizing SPSS version 22.0 software, while images were generated using GraphPad Prism version 8.0. All data are expressed as mean ± standard deviation (SD). Variance among multiple groups was analyzed using one‐way ANOVA, whereas differences between two groups were assessed through Student's *t*‐tests. A *p* value of less than 0.05 was deemed statistically significant.

## 3 Results

### 3.1 Highly Expressed JPH3 Was Associated With Poor Prognosis in TC

**FIGURE 1:**
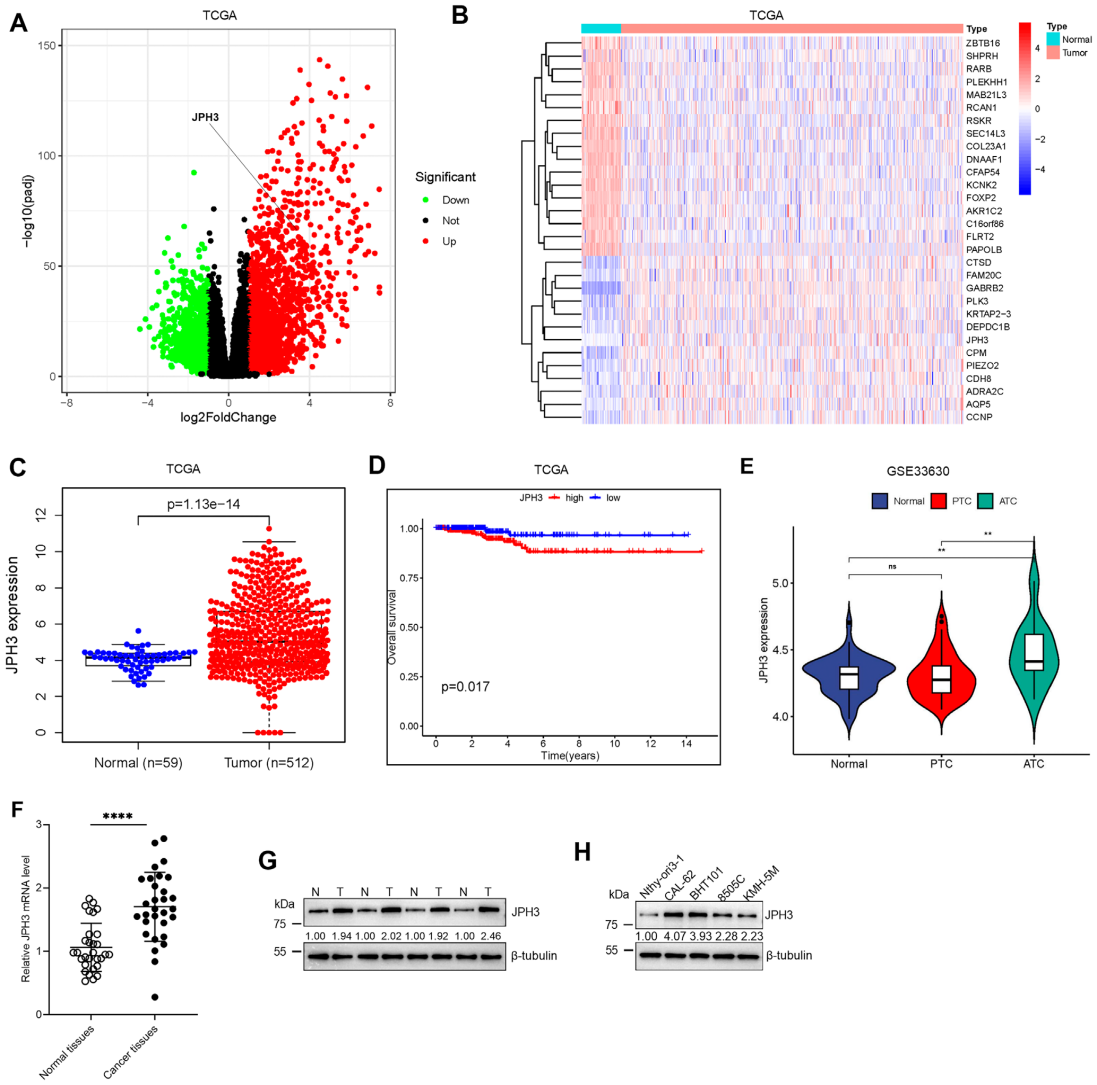
JPH3 is upregulated in TC. (A) The volcano plot delineates the genes that are differentially expressed in thyroid carcinoma, obtained from the TCGA database. (B) The heatmap presents 30 genes that demonstrate conspicuously distinct expression profiles in thyroid carcinoma, relying on the data from the TCGA database. (C) JPH3 is upregulated in thyroid carcinoma tissues compared with the normal tissues based on the TCGA database. (D) Analysis of the TCGA database demonstrates that a high expression of JPH3 is linked to poor survival prognosis in patients with TC. (E) The expression level of JPH3 in PTC and ATC through analysis of the GSE33630 dataset. (F and G) The mRNA and protein level of JPH3 in ATC tissues and their adjacent equivalents was ascertained by means of the Western blotting. (H) JPH3 expression in ATC cells and the human normal thyroid cell line Nthy‐ori3‐1 was ascertained by Western blotting. **p < 0.01, ****p < 0.0001; ns, no significant difference.

To ascertain the genes potentially implicated in TC progression, an analysis of sequencing data from the TCGA database was performed, revealing a total of 1831 upregulated and 1177 downregulated genes (Figure 1A). Notably, among the upregulated genes, JPH3 exhibited significant differences in mRNA expression when comparing normal adjacent thyroid tissues to TC tissues (Figure 1B,C). Further examination indicated that patients with elevated JPH3 mRNA levels experienced a significantly reduced survival duration compared with those with lower levels (Figure 1D). To substantiate the specific upregulation of JPH3 in ATC, we downloaded and analyzed the GSE33630 dataset. Our analysis revealed that JPH3 expression was not significantly elevated in PTC but was markedly increased in ATC (Figure 1E). Concurrently, our results confirmed that JPH3 mRNA and protein levels in TC tissues were considerably higher than those in adjacent tissues (Figure 1F,G). Additionally, JPH3 protein expression was found to be markedly elevated in four ATC cell lines (CAL‐62, BHT101, 8505C, and KMH‐5 M) relative to the human normal thyroid cell line Nthy‐ori3‐1 (Figure 1H). These findings suggest that elevated JPH3 expression may promote TC progression and diminish patient survival.

### 3.2 JPH3 Acted as an Oncogene Role in ATC Cells

**FIGURE 2:**
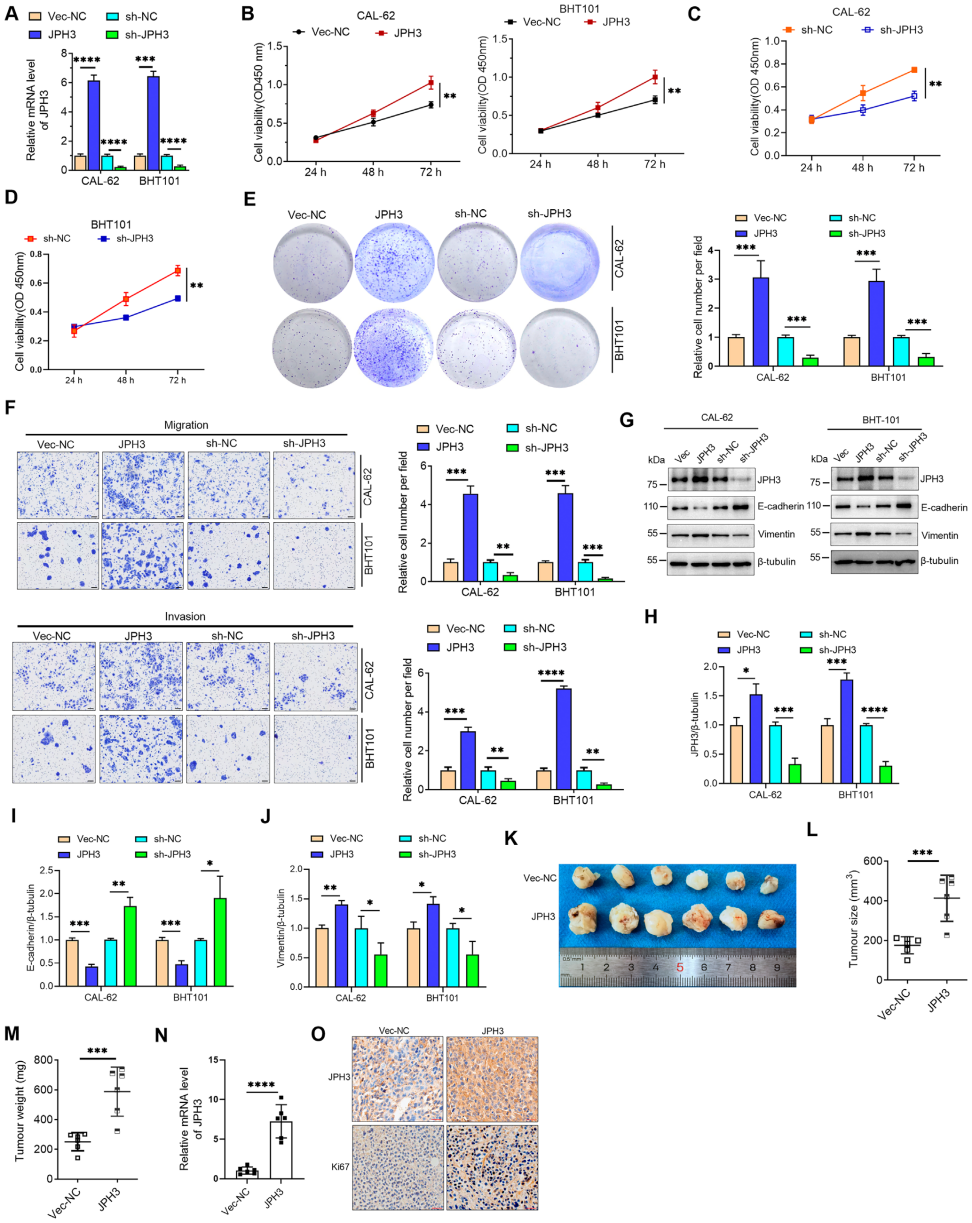
JPH3 promotes the proliferation, migration, and invasion in ATC. (A) After the transfection of JPH3 overexpression or knockdown plasmids into ATC cells for 36 h, RNA was extracted and the mRNA levels of JPH3 were detected by RT‐qPCR. (B–D) The effects of JPH3 on cell viability in ATC cells were determined by CCK‐8 assay after overexpression of JPH3 (B) and knockdown of JPH3 (C and D). (E) The effects of alterations in JPH3 levels on the colony formation of ATC cells were detected. (F) The effects of alterations in JPH3 levels on the migration and invasion of ATC cells were detected. (G) The expression levels of EMT markers were detected by Western blotting after the modulation of JPH3 expression. (H–J) The protein levels in (G) were quantified. (K) Images of subcutaneous tumors were isolated from nude mice 3 weeks after subcutaneous injection of indicated CAL‐62 cells. (L) Tumor volume was measured. (M) Tumor weight was measured. (N) The expression levels of JPH3 and Ki67 in tumor tissues were detected by IHC. (O) The mRNA level of JPH3 in tumor tissues was detected by RT‐qPCR. *p < 0.05, **p < 0.01, ***p < 0.001, ****p < 0.0001.

To further investigate the functional role of JPH3 in ATC, the expression levels of JPH3 in cells were manipulated (Figure 2A) through either overexpression or knockdown techniques. The impact of JPH3 on the cell viability of ATC cells was initially evaluated using the CCK‐8 assay, which demonstrated that JPH3 overexpression significantly enhanced the proliferation of ATC cells (CAL‐62 and BHT101), while its knockdown resulted in decreased cell viability (Figure 2B–D). Then, a colony formation assay was used to detect the proliferation ability. The results showed that JPH3 overexpression significantly enhanced the cell proliferation of ATC cells; however, the knockdown of JPH3 inhibited the cell proliferation (Figure 2E). Furthermore, we assessed the potential impact of JPH3 on the invasion and migration abilities of ATC cells through Transwell assays. The findings indicated that the overexpression of JPH3 enhanced both the invasion and migration capabilities of ATC cells, whereas the knockdown of JPH3 impeded these cellular functions (Figure 2F). The epithelial‐mesenchymal transition (EMT) is a significant biological process that plays a pivotal role in the invasion and metastasis of cancer, and it is a focal point in both biological and medical research [12, 13, 14]. Our investigations demonstrated that JPH3 overexpression led to a decrease in E‐cadherin expression while elevating Vimentin levels. Conversely, JPH3 silencing resulted in the opposite effects (Figure 2G–J).

To further explore the influence of JPH3 on the tumorigenic potential of ATC cells, we subcutaneously injected CAL‐62 cells, which stably express JPH3, into nude mice. The results indicated that the tumor growth of CAL‐62 cells with stable JPH3 expression was markedly greater in both volume and weight compared with control group cells (Figure 2K–M). Immunohistochemical staining and RT‐qPCR analyses revealed a significant increase in JPH3 levels in tumors derived from CAL‐62 cells exhibiting JPH3 overexpression (Figure 2N,O). Additionally, we confirmed that the expression of Ki67, a marker of proliferation, was significantly elevated in these tumors (Figure 2O). These findings imply that JPH3 functions as an oncogene in ATC cells.

### 3.3 JPH3 Upregulates the Expression of COL26A1 in ATC

**FIGURE 3:**
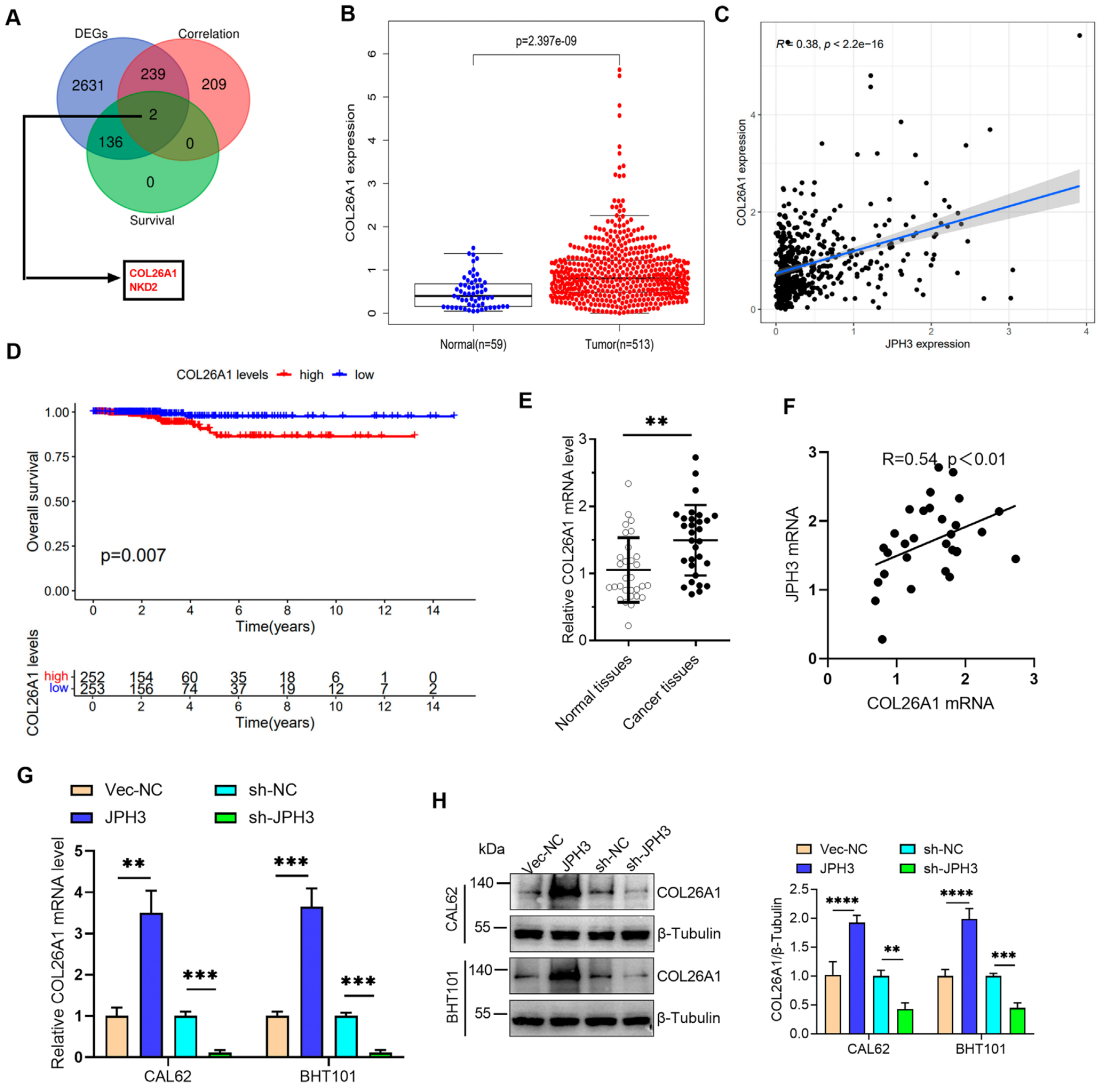
JPH3 positively regulates the expression of COL26A1 in ATC. (A) The Venn diagram analysis reveals the presence of seven overlapping genes from the TCGA database, the expression levels of which in cancer tissues and patient survival outcomes exhibit a statistically significant and positive correlation with JPH3. (B) Expression profiling of COL26A1 was conducted using the TCGA database. (C and D) Correlation analyses revealed significant associations between COL26A1 and JPH3 expression levels. (E) Kaplan–Meier survival analysis demonstrated the prognostic relevance of COL26A1 in TC. (F) RT‐qPCR assays were performed to quantify COL26A1 expression in 30 paired samples of TC tissues and adjacent non‐tumor thyroid tissues. (G) Correlation assessment between COL26A1 and JPH3 expression was carried out in 30 paired ATC and adjacent normal tissue specimens. (H) The regulatory effects of JPH3 overexpression and knockdown on COL26A1 mRNA and protein expression levels were evaluated in ATC cells. **p < 0.01, ***p < 0.001, ****p < 0.0001.

To elucidate the molecular mechanisms underlying JPH3‐mediated regulation in ATC, we analyzed significantly DEGs in thyroid cancer tissues from TCGA database. We identified genes showing significant correlation with JPH3 expression and evaluated their prognostic relevance through survival analysis. Venn diagram analysis revealed a total of two overlapping genes, COL26A1 and NKD2 (Figure 3A). To further elucidate the molecular mechanisms underlying JPH3‐mediated regulation in TC, COL26A1 was selected as a candidate gene for in‐depth investigation. Expression profiling demonstrated that COL26A1 was significantly upregulated in TC tissues (Figure 3B), with strong positive correlations observed between their expression levels and JPH3 expression (Figure 3C). However, survival analysis indicated that only COL26A1 exhibited significant associations with patient outcomes, as high expression of this gene was correlated with markedly shorter survival compared to low expression (Figure 3D). Our results revealed that COL26A1 expression was significantly elevated in 30 pairs of ATC tissues compared to adjacent normal tissues, and its expression was strongly correlated with JPH3 levels (Figure 3E,F). Furthermore, JPH3 overexpression significantly increased the mRNA level of COL26A1, whereas JPH3 knockdown led to a marked reduction in COL26A1 expression in ATC cells (Figure 3G). Then, we detected the changes in the protein level of COL26A1, which were consistent with the trend of changes in the mRNA level (Figure 3H). Collectively, these findings demonstrate that JPH3 upregulates COL26A1 expression and suggest its potential involvement in the progression of ATC.

### 3.4 JPH3 Activates the JAK–STAT Signaling Pathway in ATC Cells

**FIGURE 4:**
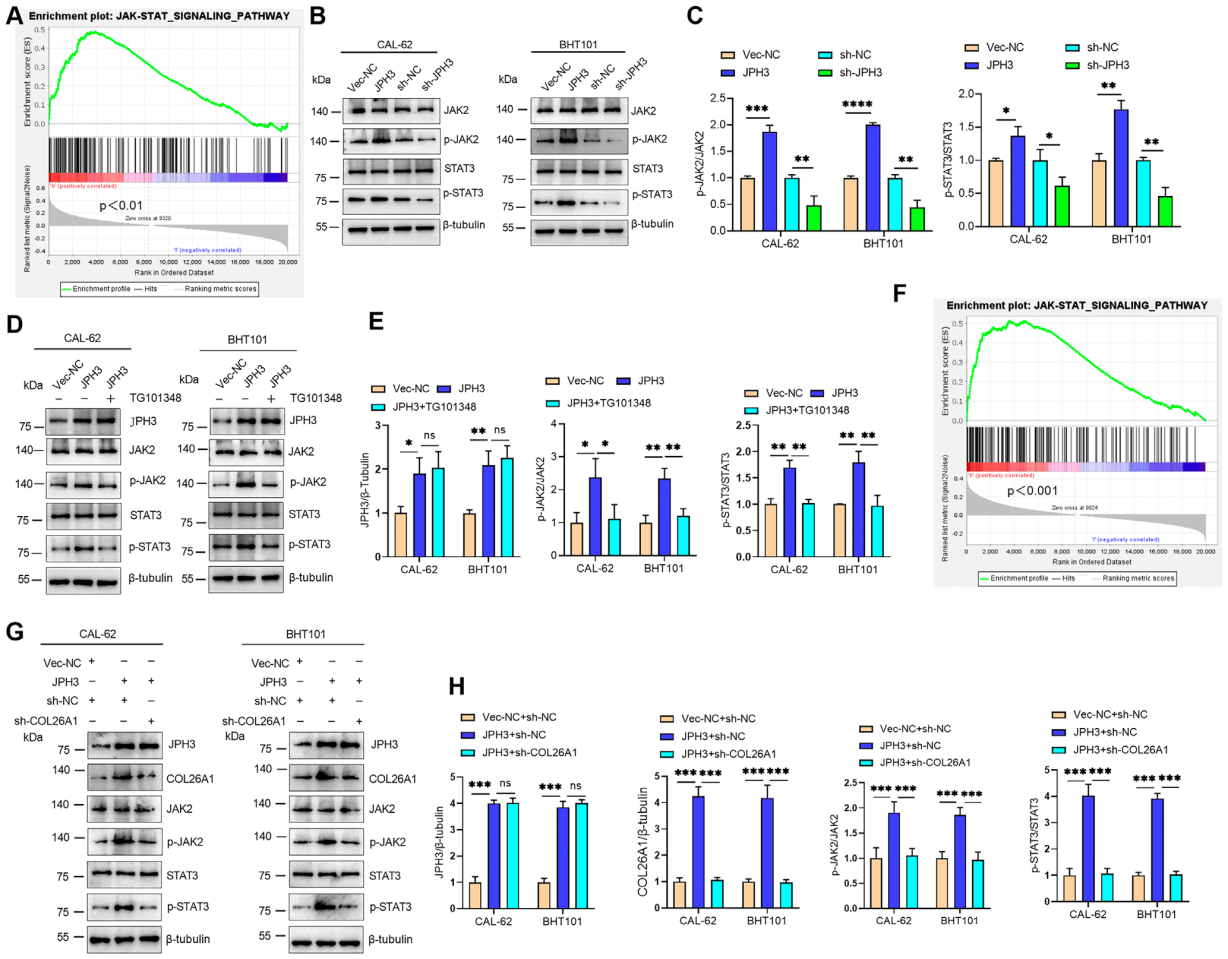
JAK–STAT signaling pathway is activated by JPH3 in ATC cells. (A) The GSEA results suggest that JPH3 might be associated with the JAK–STAT signaling pathway. (B) Western blotting was employed to determine the levels of proteins related to the JAK–STAT signaling pathway. (C) The protein levels in (B) were quantified. (D) ATC cells were transfected with Vec‐NC and JPH3 overexpression plasmids, respectively, and then treated with TG101348 as indicated. The levels of proteins related to the JAK–STAT signaling pathway were detected by Western blotting. (E) The protein levels in (D) were quantified. (F) The GSEA results suggest that COL26A1 might be associated with the JAK–STAT signaling pathway. (G) ATC cells were transfected with Vec‐NC + sh‐NC, JPH3 + sh‐NC, or JPH3 + sh‐COL26A1, respectively. The regulatory effects on key proteins in the JAK–STAT signaling pathway were assessed using Western blot analysis. (H) Quantitative densitometric analysis of the key proteins from panel (G) was performed to evaluate relative protein expression levels. *p < 0.05, **p < 0.01, ***p < 0.001, ****p < 0.0001; ns, no significant difference.

While JPH3 has been identified as an oncogene in ATC, its underlying molecular mechanism remains to be elucidated. To gain deeper insights into the relevant molecular pathways, we performed Gene Set Enrichment Analysis (GSEA) on sequencing data from thyroid cancer tissues sourced from TCGA. The results suggested a potential association between JPH3 and the JAK–STAT signaling pathway (Figure 4A). To ascertain whether JPH3 regulates this signaling cascade, we observed that JPH3 overexpression significantly elevated levels of phosphorylated JAK2 (p‐JAK2) and phosphorylated STAT3 (p‐STAT3), whereas JPH3 knockdown led to a notable decrease in these phosphorylated proteins (Figure 4B,C). Further experiments showed that TG101348, a specific JAK2 inhibitor, substantially mitigated the increase in p‐JAK2 and p‐STAT3 levels induced by JPH3 overexpression (Figure 4D,E). Our findings have demonstrated that JPH3 is capable of upregulating COL26A1 expression. However, it remained to be determined whether JPH3‐mediated activation of the JAK–STAT signaling pathway was dependent on COL26A1. Subsequent analysis revealed a significant association between COL26A1 and the JAK–STAT signaling pathway (Figure 4F). Further experimental investigations showed that COL26A1 downregulation could partially attenuate the activating effect of JPH3 overexpression on the JAK–STAT signaling pathway (Figure 4G,H). These results indicate that JPH3 is capable of activating the JAK–STAT signaling pathway through upregulation of COL26A1 in ATC.

### 3.5 JPH3 Activated the JAK–STAT Signaling to Enhance the Malignant Phenotype of ATC Cells

**FIGURE 5:**
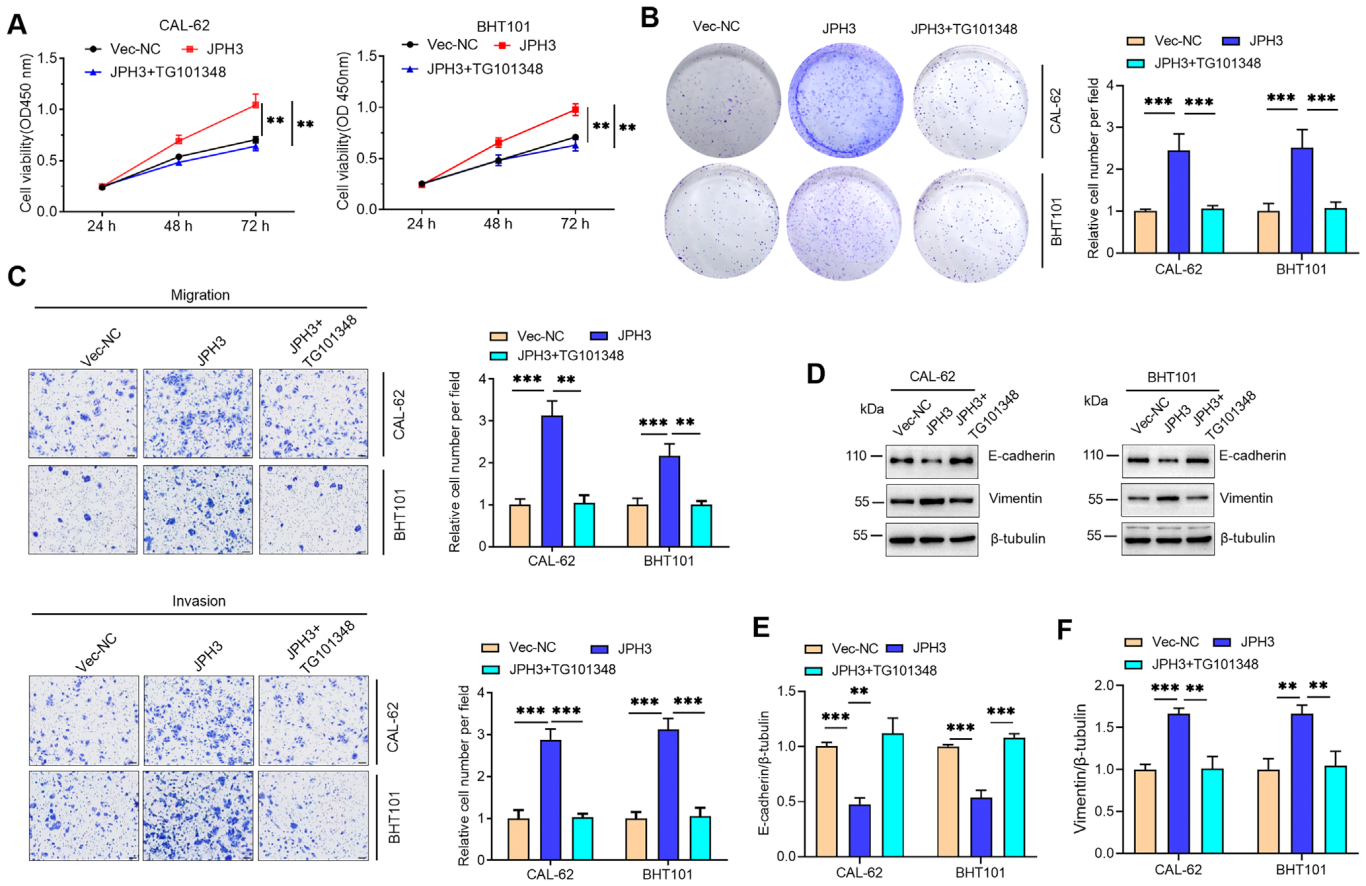
JPH3 promotes the proliferation, migration, and invasion of ATC cells through activating the JAK–STAT signaling pathway. (A) ATC cells were transfected with Vec‐NC or JPH3‐overexpressing plasmids for 24 h, then the cells were treated with TG101348 (250 nM), and CCK‐8 was used to measure the cell viability. (B) The effects of colony formation ability of ATC cells were detected after being treated with TG101348. (C) The effects of migration and invasion of ATC cells were detected after being treated with TG101348. (D) The expression levels of EMT markers were detected by Western blotting. (E and F) The protein levels in (D) were quantified **p < 0.01, ***p < 0.001.

It has been established that JPH3 activates the JAK–STAT signaling pathway; however, the precise mechanism by which JPH3 promotes proliferation, invasion, and migration of ATC cells through this pathway is not fully understood. Our research revealed that JPH3 overexpression promotes these malignant cellular behaviors. Additionally, when JPH3 overexpression was combined with TG101348 treatment, a significant reduction in the proliferation, migration, and invasion of ATC cells was observed (Figure 5A–C). This finding suggests that JPH3 facilitates these aggressive cellular traits through a mechanism involving the JAK–STAT signaling pathway. Further investigations indicated that TG101348 treatment also modulates the protein expression of EMT markers, resulting in increased E‐cadherin levels and decreased Vimentin levels (Figure 5D–F).

### 3.6 JPH3 Increased the DDP Resistance via Activating JAK/STAT3 Signaling in ATC Cells

**FIGURE 6:**
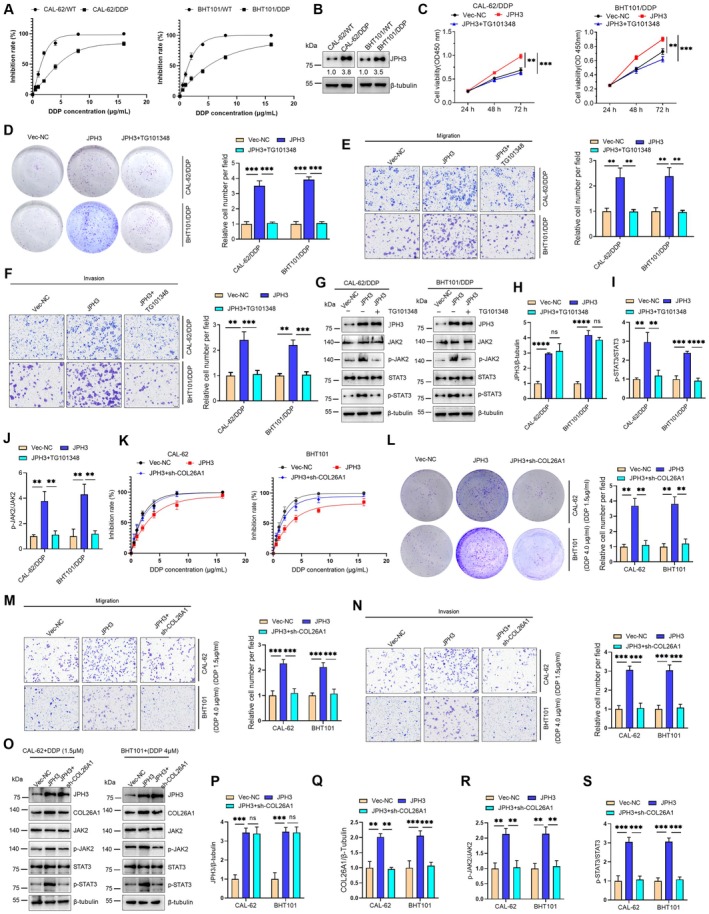
JPH3 enhances the drug resistance in DDP‐resistant ATC cells. (A) The effects of DDP on the survival of ATC/WT cells and ATC/DDP‐resistant cells. (B) The protein levels of JPH3 in ATC/WT cells and ATC/DDP‐resistant cells. (C) ATC/DDP‐resistant cells were transfected with Vec‐NC or JPH3‐overexpressing plasmids for 24 h, then the cells were treated with TG101348 (250 nM); CCK‐8 was used to measure the cell viability. (D) The effects of colony formation ability of ATC/DDP‐resistant cells were detected after treated with TG101348. (E and F) The effects of migration and invasion of ATC/DDP‐resistant cells were detected after treated with TG101348. (G) The indicated protein levels were detected by Western blotting. (H–J) The protein levels in (E) were quantified. (K) ATC cells were transfected with Vec‐NC + sh‐NC, JPH3 + sh‐NC, or JPH3 + sh‐COL26A1, respectively. The effects of DDP on the survival of indicated cells. (L) ATC cells were transfected with the indicated plasmids for 48 h, then the cells were plated into 6–well plates and cultured for 2 weeks with the indicated DDP. The effects of colony formation ability were determined. (M and N) The effects of migration and invasion of ATC cells were detected after treated with DDP. (O) The indicated protein levels were detected by Western blotting. (P‐S) The protein levels in (O) were quantified. **p < 0.01, ***p < 0.001, ****p < 0.0001; ns, no significant difference.

A resistance phenomenon to DDP has occurred in the treatment of ATC, thereby posing a certain level of challenge and difficulty for subsequent treatment [5, 15]. To determine whether JPH3 is associated with DDP‐resistant ATC, we initially selected DDP‐resistant ATC cell lines, namely CAL‐62/DDP and BHT101/DDP (Figure 6A), and identified that JPH3 was highly expressed in DDP‐resistant ATC cell lines (Figure 6B). Subsequently, we noted that the overexpression of JPH3 could significantly enhance the proliferation, invasion, and migration of DDP‐resistant ATC cells, while simultaneous treatment with TG101348 could partially counteract the promotion of the malignant cell phenotype by JPH3 (Figure 6C–F). At the molecular level, we determined that the overexpression of JPH3 in DDP‐resistant ATC cells could activate the JAK–STAT signaling pathway, while simultaneous treatment with TG101348 could inhibit the JAK–STAT signaling pathway (Figure 6G–J). Moreover, we assessed the chemosensitivity of wild‐type ATC cells to DDP by JPH3 overexpression alone or concurrent JPH3 overexpression and COL26A1 knockdown. Dose–response analysis indicated that overexpression of JPH3 made cells more resistant to DDP, while co‐knockdown of COL26A1 significantly weakened this effect (Figure 6K). Furthermore, under low‐dose DDP treatment, JPH3 overexpression promoted cell proliferation, migration, and invasion—phenotypes that were partially rescued upon COL26A1 knockdown (Figure 6L–N). Mechanistically, we demonstrated that JPH3 exerts its functional effects by upregulating COL26A1, which in turn activates the JAK/STAT signaling axis (Figure 6Q–S). These findings suggest that JPH3 can promote ATC cell resistance to DDP, and the molecular mechanism is through the activation of the JAK–STAT signaling pathway.

## 4 Discussion

ATC exhibits the traits of aggressive invasion and a high rate of distant metastasis, and it represents the type of thyroid cancer with the highest mortality rate [16]. Currently, combined chemotherapy following surgery is a commonly employed treatment modality for ATC, with DDP being extensively utilized. Nevertheless, DDP resistance frequently arises during the treatment course, resulting in a poor prognosis for patients [17]. Therefore, it is urgently necessary to explore the molecular mechanism of DDP resistance in ATC cells, which is of paramount importance for the treatment of ATC.

To tackle the problem of ATC resistance, we initially adopted bioinformatics analysis to extract information from the TCGA database and ultimately identified that JPH3 was highly expressed in TC tissues, and its high expression was associated with a poor prognosis in TC patients. Subsequently, we further validated through in vivo and in vitro experiments that JPH3 in ATC cells can boost cell proliferation, invasion, and migration, and determined that JPH3 acts as an oncogene in ATC. As of now, the role of JPH3 in tumors has received relatively scarce research. Only two reports have suggested that JPH3 serves as an anti‐oncogene to suppress tumors in digestive tract tumors and HCC, where it is expressed at a low level and has been proven to be methylated at its promoter [8, 9]. However, other researchers, via bioinformatics analysis, found that JPH3 was highly expressed in breast cancer and was related to a poor prognosis of breast cancer patients, further indicating that JPH3 might have diverse functions in tumors; that is, it could be an oncogene in certain tumors [11]. We not only made predictions through bioinformatics analysis but also further substantiated experimentally that JPH3 promotes the occurrence and development of tumors in ATC, which is in line with the prediction in breast cancer. However, existing literature indicates that JPH3 expression is downregulated in HCC, which contrasts with the findings observed in ATC [9]. Given that JPH3 promoter methylation has been implicated in its reduced expression in HCC, further investigation into the mechanisms underlying elevated JPH3 expression in ATC—such as potential differences in promoter methylation status or other regulatory pathways—may provide valuable insights into the development of DDP resistance in ATC.

**FIGURE 7:**
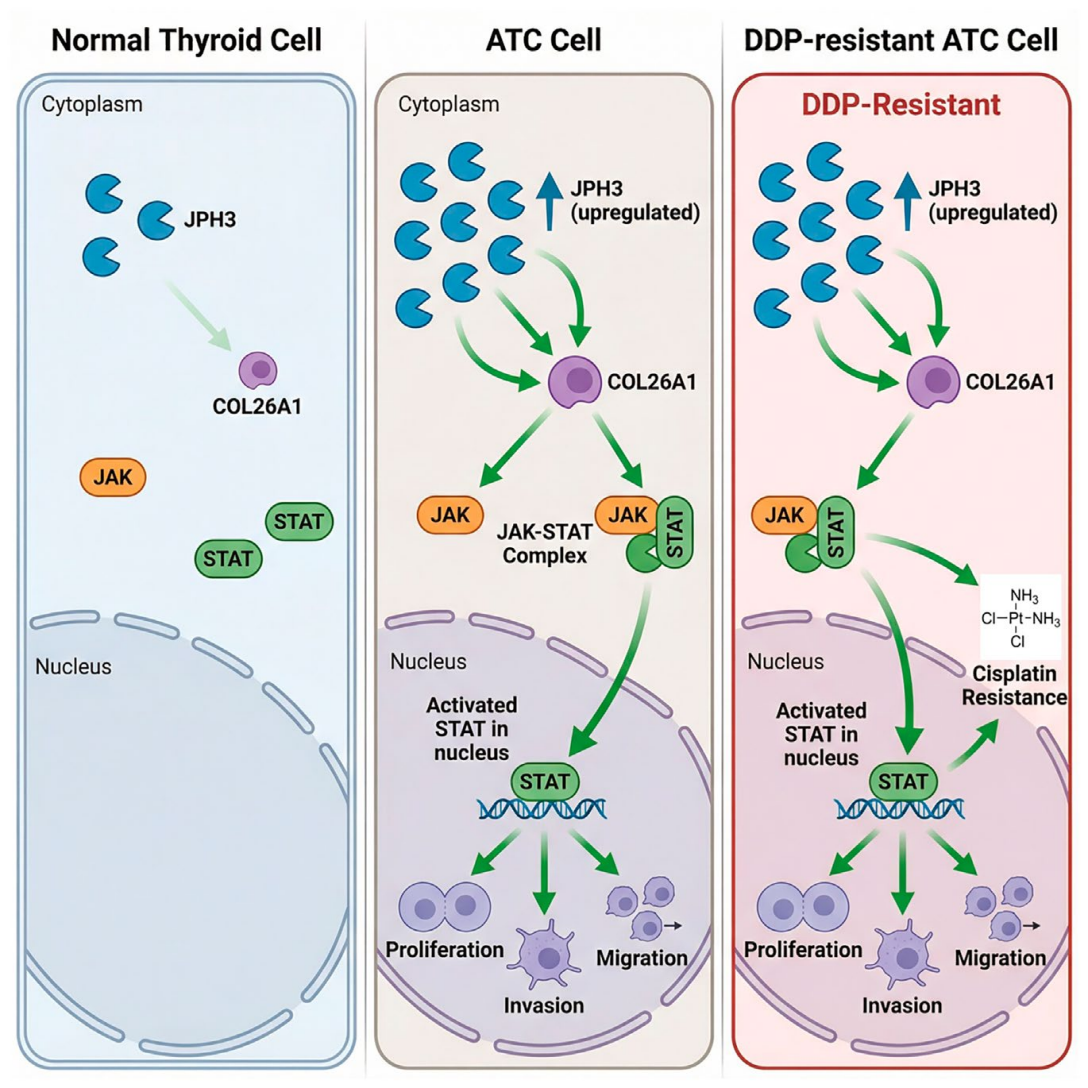
Schematic illustration of the molecular mechanism underlying JPH3‐mediated DDP resistance in ATC. JPH3 is significantly upregulated in ATC cells and promotes tumorigenesis by activating the COL26A1‐dependent JAK–STAT signaling pathway; moreover, sustained JPH3 overexpression amplifies signaling through this axis, thereby conferring resistance to DDP.

Although we have not proved the mechanism underlying the high expression of JPH3 in ATC, we have carried out in‐depth exploration of the molecular mechanism of JPH3 in ATC. For example, in terms of mechanisms, we discovered that JPH3 activates the JAK–STAT signaling pathway through upregulation of COL26A1 in ATC. Recently, COL26A1 has been identified as prognostic factors in TC [18], however, the determined mechanism remains unclear. Herein, we demonstrated COL26A1 might be an upstream activator of the JAK–STAT signaling pathway. We further confirmed that JPH3 promotes the malignant phenotype of ATC by activating the JAK–STAT signaling pathway. The JAK–STAT signaling pathway is activated in a multitude of tumors, including ATC, and is closely related to the occurrence and development of tumors [19, 20]. Therefore, our research findings suggest that JPH3 activating the JAK–STAT signaling pathway and facilitating the proliferation, invasion, and migration of ATC is highly probable. In a wide range of tumor types, the activation of the JAK–STAT pathway is able to facilitate the resistance of tumor cells to DDP, and the inhibition of this pathway can overcome tumor resistance [21, 22, 23, 24]. Nevertheless, no report has been presented concerning the relationship between the activation of the JAK–STAT signaling pathway and cisplatin resistance in DDP‐resistant ATC cells. Here, it has been demonstrated that JPH3 is highly expressed in ATC cells resistant to DDP, and the overexpression of JPH3 is capable of activating the JAK–STAT signaling pathway, thereby promoting the proliferation, invasion, and migration of DDP‐resistant ATC cells, providing a theoretical foundation for the molecular mechanism of DDP‐resistance in ATC (Figure 7).

The biological significance of JPH3 in promoting cisplatin resistance in ATC resides in its potential role as a central regulator of therapeutic resistance mechanisms. Specifically, JPH3‐mediated chemoresistance may involve the modulation of apoptotic signaling pathways, DNA damage repair processes, and drug efflux systems, implicating its involvement in key determinants of treatment failure. Future studies will further investigate the extent of JPH3's contribution to these resistance mechanisms. Furthermore, the association between JPH3 and COL26A1 points to a previously unidentified regulatory axis that may facilitate tumor adaptation to chemotherapy‐induced stress. These findings not only advance our understanding of the molecular basis of chemoresistance in ATC but also highlight JPH3 as a promising therapeutic target for overcoming drug resistance in this aggressive malignancy. It is well established that JPH3 plays a critical role in mediating drug resistance in ATC, offering both a theoretical foundation and a potential therapeutic target for understanding DDP resistance in this malignancy. However, the current study remains preliminary, with several limitations that warrant acknowledgment. Notably, the availability of clinical tissue samples from ATC patients—particularly those with DDP‐resistant disease—is limited, and comprehensive patient follow‐up data are lacking, which has constrained the scope and depth of our analyses. Consequently, further validation in larger, clinically annotated cohorts will be essential to confirm and extend these findings.

## 5 Conclusion

JPH3 is prominently expressed in thyroid carcinoma tissues, and its heightened expression correlates with poor prognostic outcomes in patients. Within the context of ATC, JPH3 acts as an oncogene, promoting tumor proliferation, invasion, and migration. Mechanistically, JPH3 may function as an oncogenic factor through the activation of the JAK–STAT signaling cascade through upregulation of COL26A1. Additional research has indicated that JPH3 enhances the resistance of ATC cells to DDP by activating this pathway, offering new perspectives on the mechanisms of ATC resistance.

## Author Contributions

Xiuyan Wang: research conception and design. Sha Luan: manuscript writing and revision. Lei Sun: data collection and analysis. Lixin Lian: data analysis and technical support. Ming Qi: final review and approval. All authors reviewed the manuscript.

## Funding

The authors have nothing to report.

## Ethics Statement

All patients provided written informed consent and voluntarily donated TC and adjacent tissue specimens. This study was approved by the Ethics Committee of Harbin First Hospital (Ethical Approval No.: Q2023‐200). The animal experiments were approved by the Animal Experimental Ethical Inspection Committee of the Fourth Clinical Hospital of Harbin Medical University (Ethical Approval No.: 2022‐DWSYLLCZ‐85).

## Conflicts of Interest

The authors declare no conflicts of interest.

## Data Availability

The data that support the findings of this study are available from the corresponding author upon reasonable request.
